# Maternal Vitamin D Status in Type 1 Diabetic Pregnancy: Impact on Neonatal Vitamin D Status and Association with Maternal Glycaemic Control

**DOI:** 10.1371/journal.pone.0074068

**Published:** 2013-09-03

**Authors:** Sarah E. Bennett, Jennifer McPeake, David R. McCance, John G. Manderson, Philip Johnston, Rachel McGalliard, Ann McGinty

**Affiliations:** 1 Nutrition and Metabolism Group, Centre for Public Health, School of Medicine, Dentistry and Biomedical Sciences, Queen’s University Belfast, Belfast, United Kingdom; 2 Regional Centre for Endocrinology and Diabetes, Royal Victoria Hospital, Belfast, United Kingdom; University of Texas Health Science Center at San Antonio, United States of America

## Abstract

**Objective:**

The first aim of this study was to assess 25-hydroxy vitamin D (25OHD) concentrations in women with type 1 diabetes (T1DM) during pregnancy, post-delivery and also foetal (cord blood) 25OHD concentrations and to examine relationships between these. The second aim of the study was to investigate potential interactions between maternal body mass index (BMI) and foetal vitamin D status. A further study aim was to examine potential relationships between maternal 25OHD and glycosylated haemoglobin (HbA_1c_) throughout pregnancy.

**Research Design and Methods:**

This was an observational study of 52 pregnant controls without diabetes and 65 pregnant women with T1DM in a university teaching hospital. Maternal serum 25OHD was measured serially throughout the pregnancy and post-delivery. Cord blood 25OHD was measured at delivery. 25OHD was measured by liquid chromatography tandem mass spectrometry (LC-MS/MS).

**Results:**

Vitamin D deficiency (25OHD <25 nmol/L) was apparent in both the T1DM subjects and controls at all 3 pregnancy trimesters. Vitamin D levels in all cord blood were <50 nmol/L. Maternal 25OHD correlated positively with cord 25OHD at all 3 trimesters in the T1DM group (*p* = 0.02; *p*<0.001; *p*<0.001). 25OHD levels within cord blood were significantly lower for women with diabetes classified as obese vs. normal weight at booking [normal weight BMI <25 kg/m^2^ vs. obese BMI >30 kg/m^2^ (nmol/L±SD); 19.93±11.15 vs. 13.73±4.74, *p* = 0.026]. In the T1DM group, HbA_1c_ at booking was significantly negatively correlated with maternal 25OHD at all 3 trimesters (*p* = 0.004; p = 0.001; *p* = 0.05).

**Conclusion:**

In T1DM pregnancy, low vitamin D levels persist throughout gestation and post-delivery. Cord blood vitamin D levels correlate with those of the mother, and are significantly lower in obese women than in their normal weight counterparts. Maternal vitamin D levels exhibit a significant negative relationship with HbA_1c_ levels, supporting a potential role for this vitamin in maintaining glycaemic control.

## Introduction

It is well established that vitamin D plays a primary role in bone health, with severe vitamin D deficiency known to cause rickets in children and osteomalacia in adults [1]. Vitamin D has also been linked to a wide variety of other non-bone health outcomes such as; cardiovascular disease, cancer, autoimmune diseases and type 1 and type 2 diabetes mellitus (T1DM, T2DM; [2]).

Vitamin D status is assessed by measuring circulating concentrations of 25-hydroxy vitamin D (25OHD). In relation to bone health, a 25OHD level of <25 nmol/l is currently defined as deficient [3]; however, the cut-off level for sufficiency remains unclear. Holick [4] suggests that for extraskeletal health, sufficiency is achieved at vitamin D levels >75 nmol/l. Hypovitaminosis D is commonly seen in both pregnant and non-pregnant women worldwide [5]; with many factors impacting on vitamin D status, including; exposure to sunlight, skin colour, season, latitude and obesity, among others [6].

In the absence of environmental and behavioural factors, 25OHD has been found to remain unchanged during pregnancy [7], while other evidence has indicated that 25OHD levels decline slightly with advancing gestation [8]. During pregnancy, 25OHD diffuses across the placental barrier, meaning the foetus relies entirely on the vitamin D status of the mother [9]. Cord concentrations are lower than maternal concentrations, therefore, if the mother is deficient so too will be the foetus [9]. Despite the lack of change/decline in 25OHD levels, levels of the biologically active vitamin D metabolite, 1,25 dihydroxy vitamin D [1,25(OH)_2_D], are known to increase by 100% or more during pregnancy [7], [10]. This rise begins at 10–12 weeks gestation and reaches a maximum in the third trimester [10]. Increased synthesis of the active vitamin D form is due to the enhanced production of 1α-hydroxylase in the decidua and placenta, as well as an oestrogen-dependent increase in vitamin D binding protein [7]. This apparent rise in 1,25(OH)_2_D has led to the suggestion that pregnant women may require higher cellular exposure to active vitamin D during the second and third trimesters, and has been interpreted by some as providing evidence for its potential role in obstetric well-being [8].

Vitamin D deficiency has been linked to multiple adverse perinatal outcomes in pregnancy including; pre-eclampsia, gestational diabetes, low birth weight, bacterial vaginosis, pre-term delivery and caesarean section [7], [11]. It has been speculated that some adverse perinatal outcomes may be due to the immunomodulatory properties of vitamin D, which may assist in the appropriate immune response to the placenta [7]. Vitamin D is also directly involved in the production of cathelicidin: antimicrobial peptides, which could potentially prevent infection during pregnancy [12].

Maternal hypovitamosis D has also been reported to be associated with a number of adverse bone and non-bone health outcomes in off-spring. There have been reports of increased incidence of rickets cases in children born to vitamin D deficient mothers [13]. The results of a large recently published prospective study has, however, found no evidence of an association between the maternal vitamin D status in pregnancy and late childhood bone mineral content (average age 10 yrs) [14]. Importantly, this cohort was largely of white European origin and therefore, less at risk of vitamin D deficiency [15]; furthermore, the authors counselled that their findings should not be taken to mean that individual 25OHD concentrations are not an important determinant of bone health, and, moreover, that their study was not designed to examine non-bone health outcomes [14]. Additionally, there is emerging evidence demonstrating an association between foetal and early childhood vitamin D deficiency and later adult disease processes, such as multiple sclerosis, cardiovascular disease, schizophrenia, certain cancers and other autoimmune diseases such as T1DM and lupus [16].

Numerous studies have established an inverse relationship between circulating levels of 25OHD and measures of adiposity [17]. The most likely mechanism underlying this, is the ability of subcutaneous fat to sequester cutaneously synthesized D_3_ [18], the 25OHD precursor. While it has been speculated that vitamin D deficiency may be causal in the development of adiposity and, potentially, its co-morbidities, a recent study using genetic markers as instrumental variables in bi-directional Mendelian randomization analysis has provided evidence that a higher BMI leads to lower 25OHD and that the likelihood of lower 25OHD increasing BMI is small [19]. These findings led the study authors to conclude that population level interventions to reduce BMI would be expected to decrease the prevalence of vitamin D deficiency. Studies in women without diabetes [20], [21] have reported significantly reduced cord 25OHD levels in women classified as obese vs. normal weight pre-pregnancy.

Women with T1DM have an increased risk of pregnancy complications including; miscarriage, congenital malformations, macrosomia, still birth, pre-term birth and pre-eclampsia [22], some of which have been reported to be associated with vitamin D deficiency in pregnancy. Further, it has been suggested that vitamin D can alter glucose and insulin metabolism, and, therefore, impact on energy available to the foetus [11]. There are numerous reports supporting a role for vitamin D in promoting insulin sensitivity and maintaining glycaemic control in T2DM, while the evidence for a similar role in T1DM is more limited.

The first aim of this observational study was to assess 25OHD concentrations in women with T1DM during pregnancy, post-delivery and also foetal (cord blood) 25OHD concentrations, and to examine relationships between these. The second aim of the study was to investigate potential interactions between maternal BMI and foetal vitamin D status. A further study aim was to examine potential relationships between maternal 25OHD and glycosylated haemoglobin (HbA_1c_) throughout pregnancy.

## Methods

### Ethics Statement

The study was approved by the Office of research Ethics Committee Northern Ireland (REC 07/NIR02/1360).

### Settings and Participants

Sixty-five pregnant women with T1DM and fifty-two pregnant controls without diabetes were recruited from the Royal Jubilee Maternity Hospital before 14 weeks of gestation. The subjects were recruited between October 1997-February 2000 [23]. All participants had a single pregnancy during the study period and all had live births and singleton pregnancies [23]. Written consent was obtained from all patients. Exclusion criteria for the control subjects were as follows: presence of glucose intolerance on routine screening [24], previous gestational diabetes mellitus, or a first-degree relative with diabetes mellitus. All T1DM subjects were confirmed as type 1 before pregnancy. All study participants were white in origin.

In all subjects, blood samples were obtained at booking and at 20, 28, 32, 36, and 38 weeks of gestation and within 48 hours post-delivery. Gestational age was estimated by ultrasound measurements and also from the date of the last menstrual period. Levels of HbA_1c_ <6.5% (47.5 mmol/mol) were considered to be normal in pregnancy. Clinical, obstetric, and neonatal descriptive variables are shown in **Table 1**. Infants were assigned to birth weight percentiles according to gestational age, sex, and current British growth standards [25]. The same approach was used to adjust neonatal BMI (weight/height^2^) for age at examination and sex. The birth weight SD score (SDS) represents the number of standard deviations (S.Ds) that the birth weight is from the mean for British births of that gestational age and sex. Preeclampsia (proteinuric pregnancy-induced hypertension) was defined using the accepted criteria of the International Society for the Study of Hypertension in Pregnancy as proposed by Davey and MacGillivray [26]. Patients with a history of hypertension, proteinuric renal disease prior to pregnancy, or who had a urinary albumin >20 g/dL or an albumin/creatinine ratio >2.0 mg/mmol recorded at less than 20 weeks gestation were excluded by definition. Neonatal anthropometric measures were performed within 12 to 72 hours of birth and included triceps and subscapular skinfold thickness.

**Table 1:** Clinical characteristics and pregnancy outcomes of study participants at baseline.

Characteristic/Outcome	Control group (n = 52)	T1DM group (n = 65)	p value
*Maternal Characteristic*			
Age (y)*	29.6±5.5	29.7±4.9	0.99
BMI at booking (kg/m^2^)*	25.7±5.4	27.6±5.2	0.07
Parity			
0	17/52 (32.7%)	28/65 (43.1%)	0.25
1	13/52 (25%)	21/65 (32.3%)	0.39
≥2	22/52 (42.3%)	16/65 (24.6%)	0.04
Previous miscarriage	11/52 (21.2%)	24/65 (36.9%)	0.10
Cigarette smoking	21/52 (40.4%)	7/65 (10.8%)	<0.001
HbA_1c_ *	4.80±0.23	6.93±0.90	<0.001
*Pregnancy outcomes*			
Preeclampsia	2/52 (3.8%)	10/65 (15.4%)	0.04
Delivery gestational age (wk)*	39.6±1.4	36.7±2.1	<0.001
Birth weight (g)*	3441±521	3338±698	0.36
Birth weight SDS*	0.1±1.07	1.35±1.18	<0.001
Neonatal BMI SDS*	−0.05±1.38	1.14±1.17	<0.001
Birth height SDS*	0.58±1.28	1.08±1.41	0.057
Birth head circumference SDS*	0.33±1.02	0.87±1.02	0.005
Tricep skinfold (cm)*	4.05±0.68	4.78±1.16	<0.001
Subscapular skinfold (cm)*	4.77±1.15	5.96±1.58	<0.001
Male sex	25/52 (48.1%)	32/65 (49.2%)	0.90
Special care baby unit admission	1/51 (2%)	28/65 (43.1%)	<0.001

* Values are expressed as means±SD.

Continuous variables were compared at baseline using independent student’s *t* test; categorical variables were compared using Chi squared test.

### Cord Blood Collection

Cord blood was collected from 56 of the babies born to women with T1DM vs. 25 from control women. Twenty millilitres of venous cord blood was taken from the cord immediately after clamping and cutting of the umbilical cord.

### Biochemical Assessment

HbA_1c_ was recorded by three different methods during the time of the study. All the previous HbA_1c_ results were adjusted to the most recent method [high-performance liquid chromatography; auto A1c HA 8140 analyzer (Arkray-Menarini Diagnostics, Berkshire, UK); intra-assay coefficient of variation, 3%; the Diabetes Control and Complications Trial aligned results reference range, <6.5%], thus allowing numeric comparison of the results.

### Total 25OHD Analysis

Levels of 25OHD_2&3_ were measured by means of liquid chromatography tandem mass spectrometry (LC-MS/MS) and results are presented as total 25OHD.

#### Standards/calibrators, quality controls, and internal standards

Working standards were prepared by diluting commercially available 25OHD_2&3_ bi-level serum controls (Chromsystems, Munchen, Germany) into horse serum (Sigma-Aldrich Co Ltd, Poole, UK). Low, medium and high quality control samples were prepared in a similar manner. Hexadeuterated analogs (d_6_-25OHD_2_; Medical Isotopes, USA; d_6_-25OHD_3_; Synthetica AS, Oslo, Norway) were diluted with 80% (v/v) methanol in isopropanol to produce final concentrations of 625 nmol/L.

#### Sample preparation

Sample preparation was performed via a liquid/liquid extraction procedure. In brief, equal amounts of d_6_-25OHD_2_ and d_6_-25OHD_3_ were added to 150 µL of each of the calibrators, controls and patient samples. Methanol was added to precipitate proteins, followed by hexane to extract vitamin D. Samples were vortex-mixed for 30 seconds and centrifuged for 5 minutes at 13,000 g. The organic layer was transferred into maximum recovery vials (Waters, UK), evaporated under nitrogen gas, and reconstituted in 75 µL of 70% (v/v) methanol in water. Following a brief vortex-mix, vials were placed into the LC-MS/MS sample manager, which was kept at 10°C, and 20 µL of each sample was injected into the system.

#### LC-MS/MS analysis

Sample analysis was performed using an automated LC-MS/MS system, which consisted of an ACQUITY UPLC® (Waters Corporation, UK) coupled to a Waters® Xevo TQ-S® tandem quadrupole mass spectrometer. Chromatographic separation of 25OHD_2&3_ and d_6_-25OHD_2&3_ was achieved using a Waters ACQUITY UPLC BEH Phenyl column (2.1×50 mm; 1.7 µm particle size), maintained at a temperature of 35°C. The mobile phases consisted of 2 mmol/L ammonium acetate (w/v) (Sigma-Aldrich Co Ltd, Poole, UK) and 0.1% formic acid (v/v) (Fisher Scientific, UK) in water (A) and methanol [LC-MS-grade; Fisher Scientific, UK (B)]. The flow rate was set to 0.450 mL/min throughout.

A Waters® Xevo TQ-S® tandem quadrupole mass spectrometer was used in positive electrospray ionisation mode. Instrument analysis time per sample was 4.5 minutes. The multiple-reaction monitoring transitions used were: *m/z* 395.4>269.3 for 25OHD_2_, *m/z* 383.3>257.3 for 25OHD_3_, *m/z* 401.3>269.2 for d_6_-25OHD_2_, and *m/z* 389.35>263.3 for d_6_-25OHD_3_. Typical elution times for 25OHD_2_, 25OHD_3,_ d_6_-25OHD_2_ and d_6_-25OHD_3_, were 2.90, 2.81, 2.89 and 2.80 minutes, respectively. System operation and data acquisition were controlled using MassLynx 4.1 software with auto data processing by the TargetLynx Application Manager.

### Statistical Analysis

A power calculation, which assumed 65 subjects in the T1DM group, and 52 subjects in the control group, and a standard deviation of 25OHD of 17 nmol/l (based upon the average of 5 measures during pregnancy across trimesters and seasons), indicated that there was over 80% power to detect a difference in mean total 25OHD of 9 nmol/L between the two groups.

Statistical analysis was carried out using the SPSS statistical software package version 18.0 (SPSS Inc., Chicago, IL, USA). Variables not normally distributed were log transformed to natural logarithms prior to analysis to allow for parametric testing. Both the control and T1DM groups were compared by independent Students *t* test or χ^2^ test, as appropriate. Intergroup comparisons in 25OHD analysis were performed with paired samples *t* test and one-way repeated measures ANOVA, as appropriate. *Post hoc* comparisons were made using Tukey to test for specific comparisons within groups between time-points. Correlations between continuous variables were assessed by Pearson’s coefficients for correlations. Multivariate models for cord blood 25OHD and HbA_1c_ were estimated based on both maternal and neonatal measures using multiple linear regression.

## Results

### Clinical Characteristics and Pregnancy Outcomes of Study Participants

Characteristics of study participants are shown in **Table 1**. Control and T1DM groups were not significantly different in age or number of previous miscarriages. BMI at booking was higher in the women with T1DM, this difference was approaching significance. While there was no significant differences between the groups when parity was <2, at a parity ≥2, the control group was significantly higher. There were significantly fewer cigarette smokers within the T1DM group. As expected, the T1DM group exhibited significantly higher HbA_1c_ levels, and also had significantly increased incidence of preeclampsia. Delivery gestational age was significantly lower for babies born to mothers with T1DM; this may explain why the rate of admission to special care baby units was significantly increased within this group. Neonatal birth weight (g) and sex did not differ between the two groups. However, tricep and subscapular skinfold thickness were both significantly greater in babies born to women with T1DM. Birth weight SDS and neonatal BMI SDS were both significantly higher in babies born to mothers with T1DM, as was birth head circumference SDS; additionally, birth height SDS was higher in these babies, this difference was approaching significance.

### Vitamin D Analysis

#### Maternal and cord blood deficiency and insufficiency

**Table 2** shows the number and percentage of pregnant women with 25OHD deficiency/insufficiency based on the current values relating to bone health. This information is presented to indicate the prevalence of vitamin D deficiency (insufficiency) in control and T1DM women according to stage of pregnancy. The results in this table have not been analysed with respect to season and observed differences in T1DM and control women are, therefore, due to differences in season of sampling.

**Table 2:** Vitamin D deficiency and insufficiency of pregnant women and neonates, split by maternal diabetes, using various cut-off levels.

	Pregnant women			
Gestation and level of vitamin D deficiency	Control group		T1DM group	
	n	%	n	%
≤14 weeks				
<12.5 nmol/l	1	4.5	1	1.8
<25 nmol/l	10	45.5	9	16.4
<50 nmol/l	16	72.7	41	74.5
<75 nmol/l	20	90.9	50	90.9
<100 nmol/l	22	100.0	54	98.2
>14<28 weeks				
<12.5 nmol/l	2	5.0	1	1.5
<25 nmol/l	21	52.5	19	29.2
<50 nmol/l	34	85.0	46	70.8
<75 nmol/l	39	97.5	61	93.8
<100 nmol/l	40	100.0	65	100.0
≥28 weeks				
<12.5 nmol/l	6	11.5	3	4.8
<25 nmol/l	26	50.0	28	45.2
<50 nmol/l	45	86.5	51	82.3
<75 nmol/l	51	98.1	62	100.0
<100 nmol/l	52	100.0	62	100.0
	**Pregnant women**			
**Neonatal level of vitamin D deficiency**	**Control group**		**T1DM group**	
	**n**	**%**	**n**	**%**
Cord blood				
<12.5 nmol/l	8	32.0	16	28.6
<25 nmol/l	23	92.0	44	78.6
<50 nmol/l	25	100.0	56	100.0

Data are cumulative n and cumulative %.

**Table 2** shows that vitamin D deficiency, defined as 25OHD levels <25 nmol/L, was apparent in both the control and the T1DM groups across the entire pregnancy. Greater than 90% of pregnant women were classified as insufficient at each time-point, regardless of whether they had T1DM or not. This meant that <10% of this pregnant population were at a sufficient level at some point throughout pregnancy.

Similar to maternal vitamin D levels, the neonates had a high incidence of 25OHD deficiency at birth, with 28–32% exhibiting severe deficiency. All babies had vitamin D levels <50 nmol/L, indicating complete lack of sufficiency within this population (**Table 2**).

#### Levels of 25OHD in T1DM subjects and controls within seasons

There was no significant difference in mean 25OHD levels between controls and women with T1DM. Also, when compared within season, levels of vitamin D were similar between the two groups, with a significant difference between the two groups only apparent in Autumn [control group vs. T1DM group (nmol/L±SD); 31.76±18.41 vs. 39.12±17.34, *p* = 0.031].

Seasonal variation in 25OHD levels was evident. As expected, Summer 25OHD levels were significantly higher than Winter and Spring in both the control [Summer vs. Winter (nmol/L±SD); 43.12±15.29 vs. 31.04±21.50, *p* = 0.043; Summer vs. Spring (nmol/L±SD); 43.12±15.29 vs. 26.55±16.53, *p* = 0.003] and T1DM [Summer vs. Winter (nmol/L±SD); 48.48±20.51 vs. 29.19±14.42, *p*<0.001; Summer vs. Spring (nmol/L±SD); 48.48±20.51 vs. 31.58±15.01, *p*<0.001] groups. Additionally, the T1DM group had evidently higher 25OHD levels in the Autumn months compared to the Winter [Autumn vs. Winter (nmol/L±SD); 39.12±17.34 vs. 29.19±14.42, *p* = 0.013]. Despite these significant seasonal differences, mean levels in both groups within each season were still below the sufficiency level of 75 nmol/L.

#### Levels of 25OHD in women with T1DM at the final stage of pregnancy, post-delivery and within cord blood

**Figure 1:**
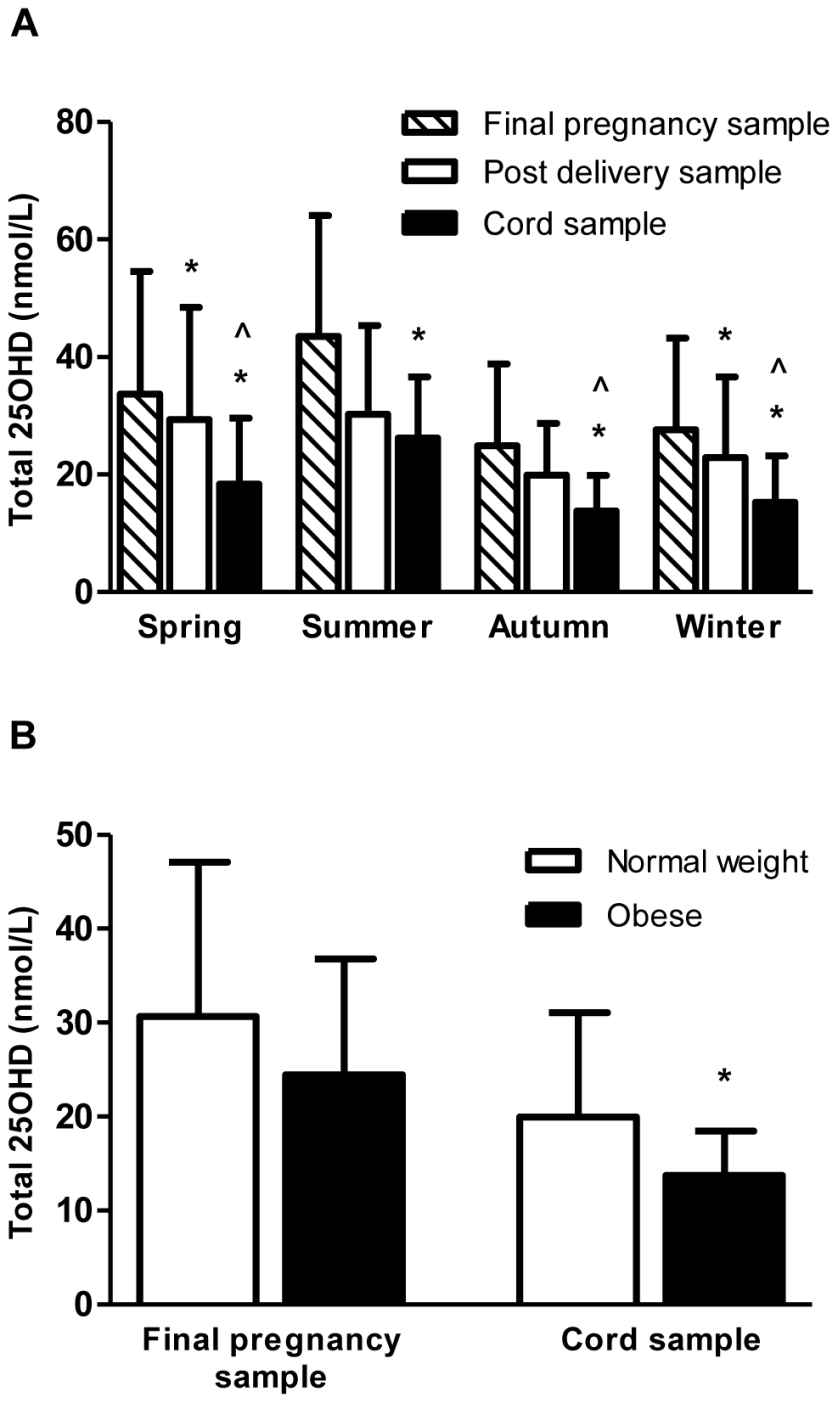
Impact of maternal vitamin D status and BMI on neonatal vitamin D levels. **(A) Within season comparisons of mean 25OHD levels (nmol/L) in final pregnancy (wk 31–38), post-delivery and cord blood samples from T1DM women and their neonates (n = 29)** Error bars indicate S.D. Comparisons were performed using paired samples *t* test: * = vs. final pregnancy sample; *p*<0.05; ? = vs. post-delivery sample; *p*<0.05. **(B) Comparison of mean 25OHD levels (nmol/L) between normal weight (BMI at booking <25 kg/m^2^; n = 22–24) and obese (BMI at booking >30 kg/m^2^; n = 16–17) T1DM women in the final pregnancy sample (wk 31–38) and neonatal cord blood.** Error bars indicate S.D. Comparisons were performed using independent student’s t-test: *p = 0.026.

Within season, 25OHD levels from the T1DM group were compared between samples taken at the final stage of pregnancy, post-delivery and from cord blood (**Figure 1A**). In Spring and Winter, 25OHD levels at the final stage of pregnancy were significantly greater than post-delivery. Further, in Autumn, Winter and Spring, there was a significant correlation between 25OHD in final pregnancy and post-delivery samples (r = 0.989, 0.913, 0.906; all p<0.001). In all seasons, 25OHD levels at the final stage of pregnancy were significantly greater than those of cord blood. Again, in all seasons there was a significant correlation between 25OHD in final pregnancy and cord blood samples (Spring r = 0.979; Summer 0.998; Autumn 0.877; Winter 0.874; all p<0.002). Furthermore, within Spring, Winter and Autumn, post-delivery 25OHD levels were significantly greater than cord blood levels.

#### Correlations between maternal serum vitamin D levels and maternal and neonatal outcomes

**Table 3** shows correlations between maternal serum 25OHD levels and maternal and neonatal outcomes. Maternal 25OHD at booking was significantly positively correlated with maternal 25OHD at trimesters 2 and 3 for both control and T1DM subjects. A similar significant positive association was seen between 25OHD at trimester 2 and trimester 3 in both groups.

**Table 3:** Maternal and cord vitamin D levels and their relationship with maternal and neonatal outcome variables.

Variable	Control Group		T1DM Group	
	*r*	*p* value	*r*	*p* value
***Maternal Vitamin D booking***				
Vitamin D trimester 2	0.92	<0.001	0.46	<0.001
Vitamin D trimester 3	0.76	<0.001	0.38	0.005
HbA_1c_ booking	−0.16	0.49	−0.38	0.004
***Maternal Vitamin D trimester 2***				
Vitamin D trimester 3	0.66	<0.001	0.64	<0.001
HbA_1c_ booking	−0.11	0.55	−0.41	0.001
HbA_1c_ trimester 2	0.06	0.76	−0.28	0.03
Birth weight (g)	0.39	0.01	0.02	0.87
Neonatal BMI SDS	0.17	0.31	−0.24	0.07
Birth height SDS	−0.06	0.73	0.23	0.08
***Maternal Vitamin D trimester 3***				
HbA_1c_ booking	−0.16	0.35	−0.25	0.05
HbA_1c_ trimester 2	−0.13	0.42	−0.22	0.08
Neonatal BMI SDS	0.11	0.43	−0.30	0.02
Birth height SDS	−0.002	0.99	0.28	0.03
***Cord Vitamin D***				
Neonatal Measure				
Neonatal BMI SDS	0.29	0.17	−0.31	0.03
Birth height SDS	−0.19	0.39	0.26	0.06
Maternal HbA_1c_				
Booking	−0.14	0.57	−0.44	0.001
Trimester 2	−0.39	0.12	−0.29	0.03
Trimester 3	−0.23	0.29	−0.22	0.12
Maternal vitamin D				
Booking	0.39	0.26	0.33	0.02
Trimester 2	0.25	0.32	0.46	<0.001
Trimester 3	0.73	<0.001	0.84	<0.001

Correlations between continuous variables were assessed by Pearson’s coefficients for correlations.

In the T1DM group, HbA_1c_ at booking was significantly negatively correlated with maternal 25OHD at all 3 timepoints. Trimester 2 HbA_1c_ was also significantly negatively associated with 25OHD at trimester 2 for the T1DM group and approaching significance at trimester 3. In the control group, birth weight (g) was significantly positively correlated with trimester 2 maternal 25OHD. The associations between maternal 25OHD and both neonatal BMI SDS and birth height SDS were approaching significance at trimester 2 and reached significance at trimester 3, in the T1DM group.

#### Correlations between cord blood vitamin D levels and maternal and neonatal outcomes

Maternal 25OHD correlated positively with cord 25OHD at all 3 trimesters in the T1DM group (**Table 3**) and at trimester 3 in the control group.

In the T1DM group, maternal HbA_1c_ at booking and trimester 2 significantly negatively correlated with cord 25OHD; as did neonatal BMI SDS. Additionally, within this group, the association between cord 25OHD and birth height SDS was approaching significance.

#### Impact of maternal BMI at booking on maternal and cord vitamin D levels in women with T1DM

In view of the trend towards a significant increase in BMI at booking in women with T1DM vs. controls (**Table 1**), the impact of maternal BMI at booking (normal weight BMI <25 kg/m^2^ vs. obese BMI >30 kg/m^2^) on maternal and cord vitamin D levels in the T1DM women was examined. Levels of 25OHD were similar at booking and trimester 2 (data not shown), and in the final pregnancy sample between the two BMI groups (**Figure 1B**). However, 25OHD levels within cord blood were significantly lower for women classified as obese at booking compared with those classified as normal weight (**Figure 1B**). Multiple linear regression analysis indicated that maternal trimester 3 vitamin D, (B = 0.462; *p*<0.001) and obese BMI at booking (B = −5.132; *p* = 0.013) significantly impacted on cord 25OHD levels. However, due to the observed independent interaction between BMI at booking and maternal trimester 3 25OHD (*p* = 0.016), the impact of these variables on cord blood 25OHD levels should be interpreted with caution. Maternal age at delivery, subscapular skinfold, triceps skinfold, SDS BMI and SDS height did not impact significantly on cord blood vitamin D levels.

#### Impact of maternal BMI at booking and maternal 25OHD on HbA_1c_ in women with T1DM

Based on the significant negative correlations noted between maternal 25OHD and HbA_1c_ at various trimesters (**Table 3**), and the significant positive correlation between maternal BMI at booking and trimester 1 HbA_1c_ (r = 0.312 and p = 0.012), the impact of maternal 25OHD and BMI at booking on HbA_1c_ in the T1DM women was examined. Multiple linear regression analysis indicated that maternal vitamin D at booking (B = −0.015; *p* = 0.011) and obese BMI at booking (B = 0.566; *p* = 0.045), both had a significant independent impact on HbA_1c_ at booking. Also, maternal trimester 2 vitamin D (B = −0.01; *p* = 0.017) independently impacted on HbA_1c_ at trimester 2.

## Discussion

The current study has provided evidence that, in T1DM pregnancy, low 25OHD levels persist throughout gestation and post-delivery. In addition, the study findings indicate that cord blood vitamin D levels correlate with those of the mother, and are significantly lower in obese women than in their normal weight counterparts. Maternal vitamin D levels were also found to exhibit a significant negative relationship with HbA_1c_ levels, supporting a role for this vitamin in maintaining glycaemic control.

The findings of the current study that, in women with T1DM, low vitamin D levels are evidenced throughout gestation are in agreement with previous groups who have reported compromised vitamin D status in pregnancy [5]. The current study found little evidence of significant differences between the control and T1DM groups during pregnancy; mean levels were similar, as were seasonal levels for Spring, Summer and Winter. A previous report has provided some evidence that pregnant women with T1DM had lower levels than pregnant women without diabetes; however, due to differences in analyses it is difficult to make direct comparisons with this study [27]. The present analysis further determined that, cord blood vitamin D levels correlated with those of the mother, and were, thus, predominantly deficient/insufficient and, in women with T1DM, low vitamin D levels persisted post-delivery. These findings support the contention that measurement of vitamin D levels prior to, and during pregnancy, should be recommended, and that vitamin D supplementation be initiated when deficiency is confirmed. Even given the lack of evidence supporting a causal link between low vitamin D and a range of non-bone health outcomes, assessment and supplementation are essential with respect to the optimal bone health of both mother and off-spring. This is particularly important for women at high-risk of deficiency [15]. Recent recommendations from the Institute of Medicine (IOM) advise pregnant and breastfeeding women in the USA and Canada to supplement with 15 µg/day to maintain optimal bone health [28]. Department of Health guidelines in the UK currently recommend pregnant women to supplement with 10 µg/day to protect against vitamin D deficiency [29], but this is disputed by some [30]. Hollis *et al*., [30] argue that supplementation of up to 100 µg/day is more effective at maintaining vitamin D sufficiency throughout pregnancy, when compared with 10 and 50 µg/day. In 2011, the UK Scientific Advisory Committee on Nutrition decided, based on new data, including the IOM report of Dietary Reference Values, that there is now sufficient evidence for an update on vitamin D, which is expected June 2014 [3].

As previously discussed, an inverse relationship between circulating levels of 25OHD and measures of adiposity has been reported, most probably due to the storage of cutaneously synthesized D_3_, the 25OHD precursor, in subcutaneous adipose tissue [17], [18]. In the current study there was an increase in BMI at booking in the T1DM group vs. control group (**Table 1**). When the impact of maternal BMI at booking on maternal and cord vitamin D levels in T1DM women was examined, maternal levels of 25OHD in women with T1DM classified as obese at booking displayed a trend for being lower. Further, 25OHD levels within cord blood were significantly lower in neonates from women classified as obese at booking compared with those classified as normal weight. Taken together, these results indicate that obese women with T1DM have lower circulating 25OHD levels and transfer reduced levels of 25OHD to their off-spring. These findings are in broad agreement with previous studies in women without T1DM [20], [21], which have reported significantly reduced cord 25OHD levels in women classified as obese vs. normal weight pre-pregnancy. While Josefson *et al*., reported an impact of maternal age and neonatal adiposity, directly measured by air displacement plethysmography, on cord vitamin D levels, in the current study, although neonatal BMI SDS was significantly negatively correlated with cord 25OHD in subjects with T1DM, regression analysis indicated that maternal age at delivery and surrogate measures of neonatal adiposity (subscapular skinfold, triceps skinfold, and SDS BMI) did not impact significantly on cord blood 25OHD.

A key finding of the current study is that in T1DM pregnancy, maternal vitamin D levels exhibit an independent inverse relationship with HbA_1c_ levels. Potential mechanisms by which vitamin D might influence glycaemic control in T1DM include, the impact of this vitamin on intracellular calcium regulation and, thus, glucose transport in target tissues, influences on insulin receptor expression and the effect of vitamin D on systemic inflammation associated with insulin resistance [31], [32]. While traditionally associated with T2DM, the importance of insulin resistance in the etiology of T1DM is being increasingly recognised, albeit that the underlying mechanisms may differ from those in T2DM [33], [34]. Low vitamin D levels are associated with insulin resistance in T2DM and its co-morbidities [35]. With respect to a causal link between vitamin D repletion and improved glucose homeostasis, limited studies have reported substantial improvements in glycaemic control (HbA_1c_ levels) in T1DM patients who achieved higher circulating 25OHD levels via supplementation [36], [37]. The results of a recent systematic review suggest a weak effect of vitamin D supplementation in reducing fasting glucose and improving insulin resistance in patients with T2DM or impaired glucose tolerance [38]. The magnitude of the reduction in fasting glucose seen in this analysis was small leading the authors to conclude that, given that HbA_1c_ did not change in patients with impaired glucose tolerance or T2DM, this is of debatable clinical significance.

One of the limitations of the current study is that it is observational. Clearly, prospective clinical randomised controlled trials of vitamin D supplementation are required to evaluate and clarify the association between vitamin D status and glycaemic control in T1DM pregnancy. A further limitation is that information is not available on dietary or supplemental vitamin D intake, in these women. However, the low levels of vitamin D found in both the control and T1DM groups would appear to argue against the widespread use of augmented intake.

In conclusion, the finding that vitamin D dependent processes may be negatively impacted by maternal nutritional status and obesity in a T1DM cohort is a significant one and provides further evidence that population-based interventions aimed at reducing obesity and improving pre-pregnancy and gestational vitamin D status are needed. Given the safety of vitamin D, the low cost of supplementation and its requirement for optimal bone health (and potentially other health outcomes) in both the mother and off-spring, the findings of the current study support the case for determining vitamin D status and, if necessary, administering vitamin D in T1DM pregnancy.
